# Stimulation of AMPK Prevents Diabetes-Induced Photoreceptor Cell Degeneration

**DOI:** 10.1155/2021/5587340

**Published:** 2021-05-13

**Authors:** Shiyu Song, Shuyin Bao, Chenghong Zhang, Jinwei Zhang, Jiajun Lv, Xiaoyu Li, Maryam Chudhary, Xiang Ren, Li Kong

**Affiliations:** ^1^Department of Histology and Embryology, College of Basic Medicine, Dalian Medical University, Dalian, 116044 LiaoNing Province, China; ^2^Inner Mongolia University for Nationalities, 028300 Tongliao, Inner Mongolia, China

## Abstract

Diabetic retinopathy (DR) is a kind of severe retinal neurodegeneration. The advanced glycation end products (AGEs) affect autophagy, and mitochondrial function is involved in DR. Adenosine-activated protein kinase (AMPK) is an important metabolic sensor that can regulate energy homeostasis in cells. However, the effect of AMPK in DR is still not fully understood. In this study, we investigated the effect of AMPK on diabetes-induced photoreceptor cell degeneration. *In vivo*, a diabetic mouse model was established by streptozotocin (STZ) injection. Haematoxylin-eosin (HE) staining was used to observe retinal morphology and measure the thicknesses of different layers in the retina. Electroretinogram (ERG) was used to evaluate retinal function. *In vitro*, 661w cells were treated with AGEs with/without an AMPK agonist (metformin) or AMPK inhibitor (compound C). Flow cytometry and CCK-8 assays were used to analyse apoptosis. Mitochondrial membrane potential was analysed by JC-1. Western blotting and qRT-PCR were used to examine the expression of related proteins and genes, respectively. The wave amplitude and the thickness of the outer nuclear layer were decreased in diabetic mice. The expression of rhodopsin and opsin was also decreased in diabetic mice. *In vitro*, the percentage of apoptotic cells was increased, the expression of the apoptosis-related protein Bax was increased, and Bcl-2 was decreased after AGE treatment in 661w cells. The expression of the autophagy-related protein LC3 was decreased, and p62 was increased. The mitochondrial-related gene expression and membrane potential were decreased, and mitochondrial morphology was abnormal, as observed by TEM. However, AMPK stimulation ameliorated this effect. These results indicate that AMPK stimulation can delay diabetes-induced photoreceptor degeneration by regulating autophagy and mitochondrial function.

## 1. Introduction

Diabetes mellitus has become a major public health problem in the world [1] and is a glucose metabolism disorder characterized by hyperglycaemia. Diabetes is associated with complications that affect many organs, such as the heart, brain, kidney, peripheral nerves, and eye [2].

Diabetic retinopathy is one of the major complications of diabetes, and it is also the main cause of vision loss. A large number of studies have shown that diabetic retinopathy can affect retinal neurons [3]. Studies have shown that this effect may be due to neuronal dysfunction caused by advanced glycation end products (AGEs) [4, 5], which can inhibit autophagy in cells [6]. Autophagy is a conserved and important catabolic pathway in all nucleated cells [7, 8] that generally maintains the stability of the intracellular environment through lysosomal degradation and cellular component recycling [9]. Normal autophagy is generally considered to promote survival. Recent evidence shows that AGEs can inhibit normal autophagy [10], which may lead to cellular dysfunction and apoptosis.

In addition, mitochondrial function is closely associated with diabetic retinopathy [11]. Mitochondria are critical for energy production for metabolic activities, and their main functions include redox balance [12]. Changes in mitochondrial function and oxidative stress are increased in a high glucose environment. Research on visual impairment caused by retinal diseases and hereditary mitochondrial diseases further illustrates the importance of normal mitochondrial function in the diabetic retina [13].

Adenosine-activated protein kinase (AMPK) is a heterotrimeric protein kinase that consists of a catalytic subunit (*α*) and two regulatory units (*β* and *γ*). AMPK is an important metabolic sensor that can regulate the energy homeostasis of cells. AMPK is also closely associated with mitochondrial function and regulates autophagic degradation [14], which helps to maintain cellular homeostasis and reduce apoptosis and plays an important role in neurodegenerative diseases [15].

Upregulation of AMPK can prevent various eye diseases, including oxidative damage to human retinal pigment epithelial (RPE) cells [5], cataracts [6], and optic neuritis [16]. However, the specific role of AMPK in diabetes-induced photoreceptor degeneration is still unclear.

Therefore, in this study, we used metformin, which is a widely used AMPK activator, to stimulate AMPK *in vitro* and explore the effect of AMPK on diabetes-induced photoreceptor degeneration and the possible specific mechanism. This study was aimed to provide an effective basis for new clinical therapeutic targets for DR.

## 2. Materials and Methods

### 2.1. Cell Culture and Regents

The mouse photoreceptor-derived (661w) cell line was obtained from Professor Muayyad R. AI-Ubaidi of the University of Oklahoma. The cells were cultured in DMEM (Gibco) containing 10% foetal bovine serum (Biological Industries) and 1% antibiotics (100 units/ml penicillin and 100 *μ*g/ml streptomycin) (HyClone) in a humidified 5% CO_2_ atmosphere at 37°C. The medium was replaced every 1 or 2 days. The cells were washed with PBS (Solarbio) before the experiments. For AGE treatment (Bioss, China, catalogue number: bs-1158P), AGEs were a white powder sourced from glycated BSA (purity: 98%). AGEs were dissolved at a concentration of 5 mg/ml in DMEM without foetal bovine serum and stored at -20°C. Metformin (Sigma) was dissolved at concentration of 50 mM in PBS and stored at -20°C. Compound C(MedChemExpress, USA) was dissolved at 1 mM in DMEM without foetal bovine serum and stored at 4°C.

### 2.2. Research Design

Streptozotocin (STZ) was injected intraperitoneally into mice to induce a diabetic mouse model. We divided the animals into a control group and a diabetes group. *In vitro*, the cells were divided into the control group, AGE group, AGE+Met group, and AGE+Met+compound C group.

### 2.3. Diabetes Model Establishment

All experimental animal procedures were conducted in accordance with the institutional guidelines for the care and use of laboratory animals, and the experimental program was approved by the Institutional Animal Care and Use Committee of Dalian Medical University Laboratory Animal Centre. All experiments were performed with 4- to 6-week-old male C57BL/6J mice. Animals were housed in an air-conditioned environment with a 12-h light-dark cycle and had free access to water and food for 1 week. Then, the mice were randomly divided into a control group and a diabetes model group. The diabetic group was administered an intraperitoneal injection of STZ (50 mg/kg) after 12 h of fasting once a day for 5 consecutive days. Blood was collected from each mouse's tail, and when the random blood glucose was greater than 16.7 mmol/L, the establishment of the diabetes model was confirmed to be successful [17]. The control group was intraperitoneally injected with an equal volume of sodium citrate buffer.

### 2.4. Cell Counting Kit-8 Assay

We performed a Cell Counting Kit-8 (CCK-8; Bioss, China) assay according to the manufacturer's instructions to measure cell viability. A total of 5 × 10^3^ cells were added to each well of a 96-well plate (Guangzhou Jet Bio-Filtration Co., Ltd) and underwent different treatments. In brief, the medium was aspirated, and a mixture of the CCK-8 reaction solution and new serum-free medium at a 1 : 10 ratio was added to the 96-well plate and incubated at 37°C for 2 h in the dark. The absorbance was measured at 450 nm with a microplate reader (Varioskan Flash, Thermo Fisher).

### 2.5. Western Blot Analysis

Equal amounts of protein were separated by sodium dodecyl sulfate-polyacrylamide gel electrophoresis (SDS-PAGE) using 12% polyacrylamide gels. The resolved proteins were electroblotted onto Immobilon polyvinylidene difluoride membranes (Millipore, Bedford, MA, USA) for western blot analysis. Primary antibodies against AGEs (Abcam, ab23722, 1 : 2000), AMPK (ProteinTech, 66536-1-Ig, 1 : 1000), p-AMPK (Cell Signaling Technology, 2535 T, 1 : 1000), LC3 (ProteinTech, 14600-1-AP, 1 : 1000), p62 (ProteinTech, 18420-1-AP, 1 : 1000), Bax (ProteinTech, 23931-1-AP, 1 : 1000), Bcl-2 (ABclonal, A0208, 1 : 1000), and *β*-actin (Cell Signaling Technology, 4970S, 1 : 2000) were used according to the manufacturer's instructions. After the membranes were incubated overnight at 4°C, the membranes were washed three times with 1x TBST for 15 minutes. Subsequently, the membranes were incubated with goat anti-rabbit IgG (Bioss, bs-0295G-HRP, 1 : 2000) and goat anti-mouse IgG (Bioss, bs-0296G-HRP, 1 : 2000) for 1.5 h at room temperature and then washed 3 times with 1x TBST for 15 minutes. Finally, the membranes were exposed to X-ray film using an enhanced chemiluminescence system. The intensities of the bands were measured using the LabWorks 4.5 software.

### 2.6. Flow Cytometry

Annexin V/PI and binding buffer (KeyGEN Biotech) were used to determine the apoptotic rate. 661w cells were treated as indicated and washed with PBS three times before apoptosis analysis. After being collected and resuspended in 500 *μ*l of 1x binding buffer, the cells were stained with Annexin V/PI and incubated in the dark for 15 minutes at room temperature. Finally, the samples were mixed and analysed on the instrument.

### 2.7. Quantitative Real-Time Polymerase Chain Reaction (qRT-PCR)

According to the manufacturer's instructions, total RNA was extracted from treated 661w cells with TRIzol (Takara). GAPDH was used as a housekeeping loading control. The primers sequences were as follows: PGC-1*α* (F: 5'-GCACCAGAAAACAGCTCCAAG-3' and R: 5'-CGTCAAACACAGCTTGACAGC-3'); NRF-1 (F: 5'-GAGACGCTGCTTTCAGTCCTT-3' and R: 5'-TGGGCTTCTATGGTAGCCATGTGT-3'); Tfam (F: 5'-ATTCCGAAGTGTTTTTCCAGCA-3' and R: 5'-TCTGAAAGTTTTGCATCTGGGT-3'); SOD2 (F: 5'-GAACAACAGGCCTTATTCCGC-3' and R: 5'-ACAGCACCCCAGTCATAGTG-3'); and GAPDH (F: 5'-TGTGATGGGTGTGAACCACGAGAA-3' and R: 5'-GAGCCCTTCCACAATGCCAAAGTT-3'). The conditions of the qRT-PCR amplification were as follows: 94°C for 30 s, 94°C for 5 s, 55°C for 15 s, and 72°C for 10 s for 40 cycles. The data were analysed by the 2(–Delta Delta C(T)) method.

### 2.8. Mitochondrial DNA Copy Number

The total DNA was extracted from 661w cells with DNA extraction kit (Solarbio, D7100). The specific primer sequences of mtDNA were as follows: F: 5'-TACCAAGGCCACCACACTCCTATT-3' and R: 5'-AAATTCCTGTTGGAGGTCAGCAGC-3'. The specific primer sequences of *β*-actin gene in nuclear DNA were as follows: F: 5'-TCGTACCACAGGCATTGTGATGGA-3'and R: 5'-TGATGTCACGCACGATTTCCCTCT-3'. The reaction cycle program is setting as follows: 94°C for 30 seconds, followed by 40 cycles of 94°C for 5 seconds, 55°C for 15 seconds, and 72°C for 10 seconds. The mtDNA/*β*-actin ratio was calculated for each specimen as an index of the relative content of mtDNA.

### 2.9. Transmission Electron Microscopy (TEM)

The cells were treated as indicated, collected, and fixed at 4°C with glutaraldehyde. The samples were dehydrated using an ethanol series. Ultrathin slices were prepared and observed under a transmission electron microscope (JEOL JEM-1400).

### 2.10. Electroretinogram(ERG)

The ERG was recorded after the establishment of the diabetic mouse model. The mice were acclimated in darkness overnight before ERG measurement. Then, the mice were anaesthetized by an intraperitoneal injection of 1% pentobarbital sodium (7.5 *μ*l/g) in the dark, and the pupils were dilated with 0.5% tropine and 0.5% phenylephrine. The anaesthetized mice were placed on the platform, the recording ring electrode was placed in the centre of the corneal surface, and the flash electroretinogram was recorded. The grounding electrode was connected to the hind limb, and the negative electrode was placed on the forehead. The ERG was recorded at 3 cd.s/m2 flash intensity after light stimulation in the different experimental groups, and the functional changes in photoreceptor cells were analysed according to the amplitudes of the a wave and b wave.

### 2.11. Determination of Mitochondrial Membrane Potential

Changes in the mitochondrial membrane potential were examined by staining cells with the cationic dye JC-1. After being incubated with JC-1 (Beyotime, C2006) staining solution at 37°C in an incubator for 15 minutes, 661w cells were washed with JC-1 staining buffer 2 times. After incubation, DMEM without foetal bovine serum was added, and the cells were photographed under a fluorescence microscope. Statistical analysis was performed using the IPP software.

### 2.12. Morphological Analysis

Eyeball samples were fixed in Bouin's fluid for 24 h and then in 70% ethanol for 24–60 h. After being dehydrated in an ethanol gradient, the eyeballs were embedded in paraffin and cut into 5-*μ*m-thick sections. Paraffin-embedded sections containing the optic nerve were reserved for HE staining. Microstructural retinal changes were examined under a microscope (Nikon ECLIPSE 80i).

### 2.13. DAPI Staining

661w cells were resuspended and seeded on glass coverslips. After the different treatments, the cells were fixed with a 4% formaldehyde solution, and then, the cells were subjected to antigen retrieval with 0.5% Triton X-100 (Sigma Aldrich, USA). Subsequently, the nuclei were stained with DAPI, and the cells were covered with coverslips. Images were taken using a fluorescence microscope and analysed by software (Nikon Ti-S, Japan) (×400).

### 2.14. Statistical Analysis

Statistical analyses were conducted using the SPSS version 18.0 software (SPSS Inc., Chicago, IL, USA), and the results are depicted as the mean ± SD. The differences among groups were determined via one-way analysis of variance and *t*-tests. A *P* value < 0.05 was considered significant.

## 3. Results

### 3.1. Diabetes Induced Photoreceptor Cell Degeneration and Related Factors

**Figure 1:**
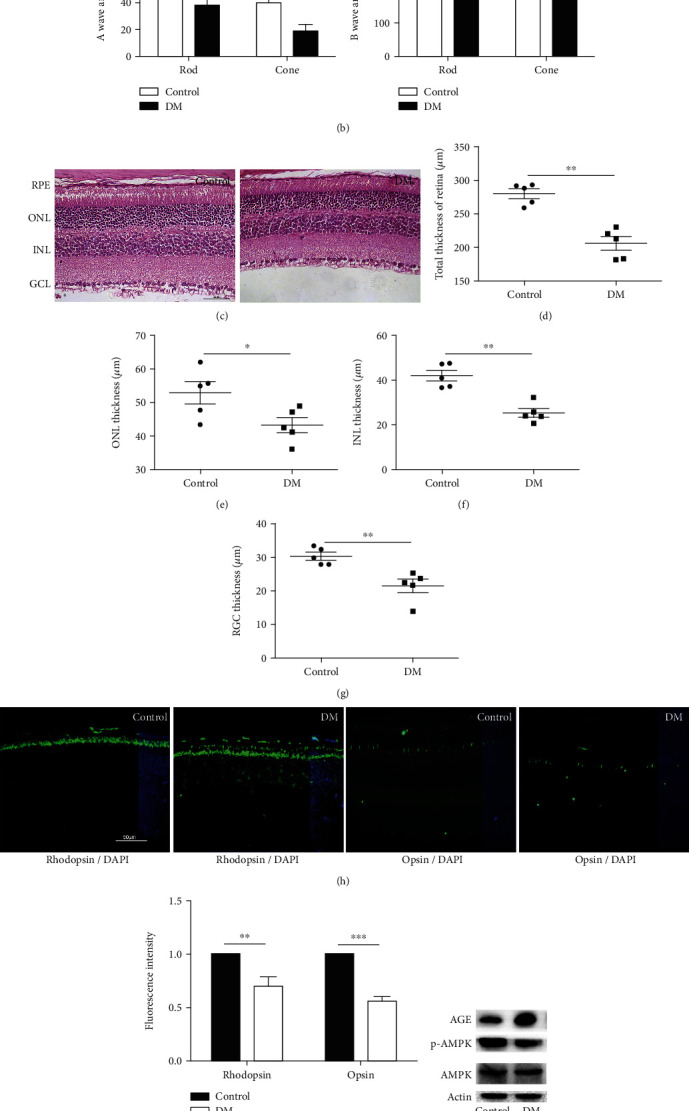
Diabetes induced photoreceptor cell degeneration *in vivo*. (a, b) Retinal function in diabetic mice was measured by ERG. (c) Retinal morphology in diabetic mice was observed by HE staining. The thicknesses of the total retina (d), ONL (e), INL (f), and RGC (g) were measured in diabetic mice. (h, i) The expression of rhodopsin and opsin was measured in diabetic mice. (j, l) The expression of AGEs and p-AMPK as measured in diabetic mice. The data are presented as the mean ± SD (*n* = 5 in each group). ^∗^*p* < 0.05, ^∗∗^*p* < 0.01, ^∗∗∗^*p* < 0.001.

The ERG waveform (Figure 1(a)) and the statistical results (Figure 1(b)) showed that the peak values of the a and b waves of rod and cone photoreceptor in diabetic mice were decreased compared with those of control mice. We also used HE staining to observe the morphology and measure the thicknesses of the different layers in the retina. The results showed that the thicknesses of the total retina, ONL, INL, and GCL in diabetic mice were decreased compared with those in control mice (Figures 1(d)–1(g)). The immunofluorescence results showed that the distribution of rhodopsin in the retina in diabetic mice was obviously disordered, and the expression level of rhodopsin was decreased. Moreover, the expression of opsin was decreased in diabetic mice compared with control mice (Figures 1(h) and 1(i)). The western blot results showed that AGE expression in diabetic mice was increased significantly, while the expression of p-AMPK was decreased (Figures 1(j)–1(l)). These results indicated that diabetes-induced photoreceptor degeneration was associated with increased AGEs and decreased AMPK.

### 3.2. Stimulation of AMPK Prevented Diabetes-Induced Photoreceptor Cell Degeneration In Vitro

**Figure 2:**
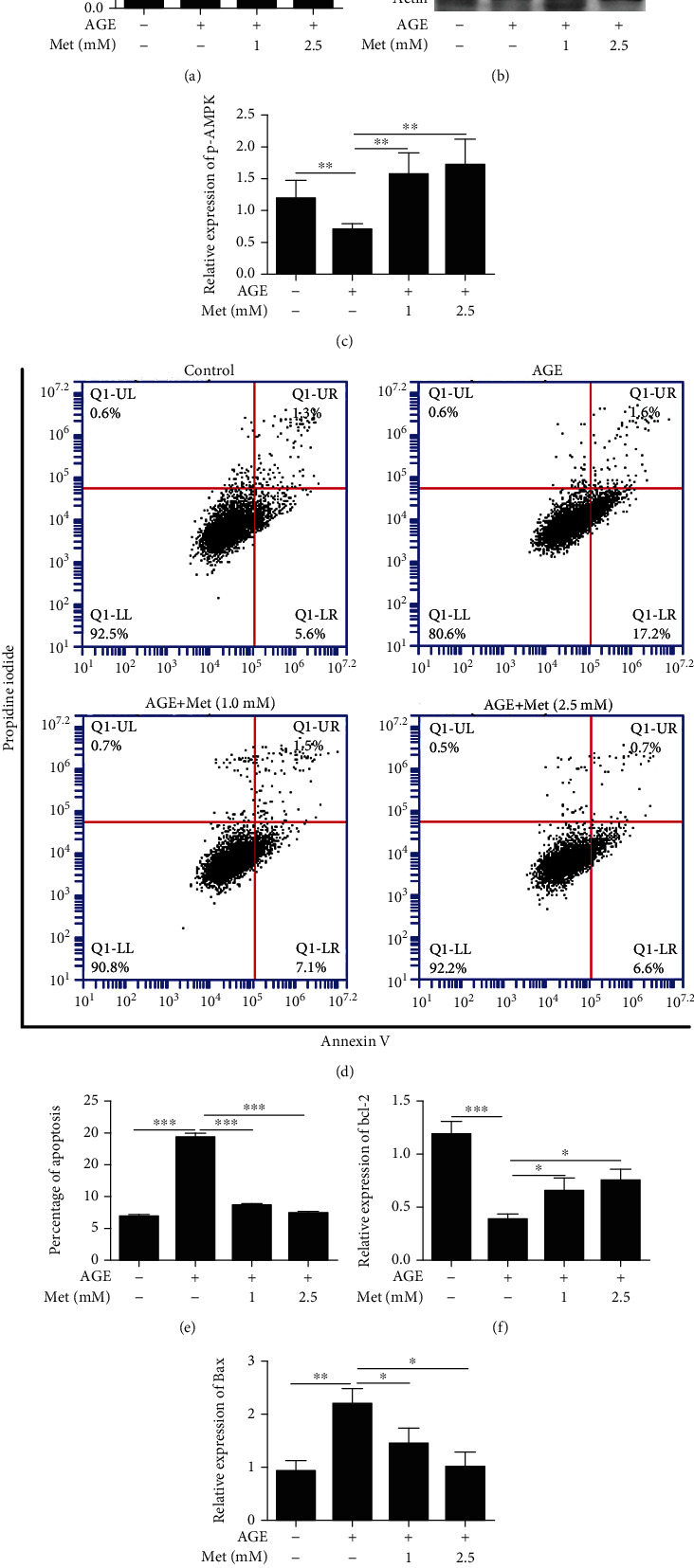
AMPK stimulation inhibited AGE-induced 661w cell apoptosis. (a) The viability of 661w cells was measured by CCK-8 assays after AGE and metformin treatment. The effect of AMPK stimulation on the expression of p-AMPK (b, c), Bcl-2 (b, f), and Bax (b, g) after AGE treatment. (d, e) The effect of AMPK stimulation on AGE-induced 661w cell apoptosis was examined by flow cytometry. The data are expressed as the mean ± SD (*n* = 3 for each group). ^∗^*p* < 0.05, ^∗∗^*p* < 0.01, ^∗∗∗^*p* < 0.001.

*In vitro*, 661w cells were treated with AGE with different concentrations of metformin, and we determined 661w cell viability by CCK-8 assays. The data showed that viability decreased after AGE treatment, but it can be increased after metformin treatment compared with only AGE treatment (Figure 2(a)). Subsequently, we examined the effect of AMPK stimulation on AGE-induced apoptosis in 661w cells. The western blot results showed that the expression of p-AMPK was increased significantly after treatment with different concentrations of metformin (an AMPK agonist) (Figures 2(b) and 2(c)) compared with AGE treatment alone. Moreover, the expression of the apoptosis-related protein bcl-2 increased and Bax decreased compared with that of AGE treatment alone (Figures 2(b), 2(f), and 2(g)). In addition, flow cytometry showed that the percentage of apoptotic cells was increased in the AGE-treated group compared with the control group; however, AMPK stimulation inhibited this effect (Figure 2(d)).

### 3.3. Stimulation of AMPK Prevented Diabetes-Induced Photoreceptor Cell Degeneration Associated with the Regulation of Mitochondrial Function and Autophagy

**Figure 3:**
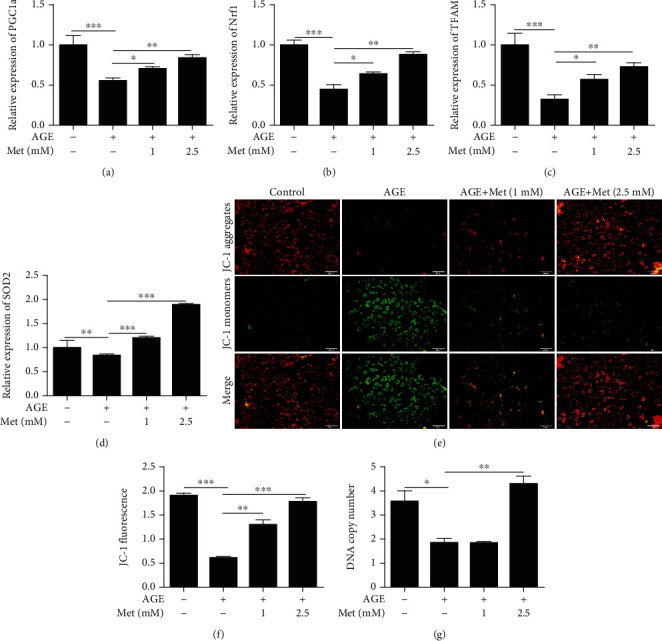
AMPK stimulation prevented diabetes-induced photoreceptor cell degeneration through the regulation of mitochondrial function. The expression of PGC1*α* (a), Nrf1 (b), Tfam (c), and SOD2 (d) and the DNA copy number (g) in 661w cells treated with AGEs after AMPK stimulation were measured by qRT-PCR. (e, f) The effect of AMPK stimulation on the mitochondrial membrane potential in 661w cells after AGE treatment. The data are expressed as the mean ± SD (*n* = 3 for each group). ^∗^*p* < 0.05, ^∗∗^*p* < 0.01, ^∗∗∗^*p* < 0.001.

We used qRT-PCR to measure the expression of genes associated with mitochondrial biogenesis and oxidative defence. We measured the expression of peroxisome proliferator-activated receptor gamma coactivator 1 alpha (PGC1*α*), mitochondrial transcription factor A (Tfam), nuclear respiratory factor 1 (Nrf1), and superoxide dismutase 2 (SOD2). The expression levels of PGC1*α*, Nrf1, Tfam, and SOD2 were decreased after AGE treatment; however, the expression levels of these genes were increased after AMPK stimulation compared with AGE-only treatment (Figures 3(a)–3(d)). The increased expression of Nrf1, Tfam, PGC1*α*, and SOD2 suggested an increase in mitochondrial function. To evaluate this possibility, we measured mitochondrial DNA content relative to nuclear DNA content by qPCR and analysed mitochondrial membrane potential by JC-1 assays. The mitochondrial DNA copy number was increased significantly after AMPK stimulation (Figure 3(g)). Moreover, the mitochondrial membrane potential assay results showed that the red/green fluorescence ratio was decreased after AGE treatment in 661w cells; however, the effect was abrogated by AMPK stimulation (Figures 3(e) and 3(f)).

**Figure 4:**
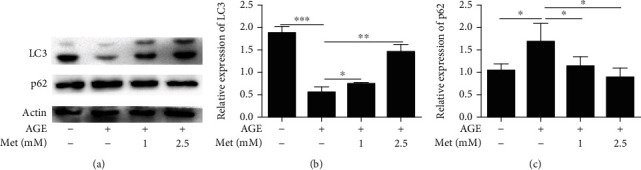
AMPK stimulation prevented diabetes-induced photoreceptor cell degeneration via autophagy regulation. Western blotting (a) was used to evaluate the expression levels of LC3 and p62 in 661w cells treated with AGEs after AMPK stimulation. Densitometric analysis showed that the elevation in LC3 (b) and p62 (c) was statistically significant. The data are expressed as the mean ± SD (*n* = 3 for each group). ^∗^*p* < 0.05, ^∗∗^*p* < 0.01, ^∗∗∗^*p* < 0.001.

In addition, we also measured the expression of LC3 and p62, which are biomarkers of autophagy. The western blot results showed that the expression of LC3 was significantly decreased, while the expression of p62 was significantly increased after AGE treatment compared with those in the control group. However, the expression of LC3 was significantly increased, and the expression of p62 was significantly decreased after AMPK stimulation compared with AGE-only treatment (Figures 4(a)–4(c)). These results indicate that the AMPK pathway may play an important role in regulating mitochondrial function and autophagy in diabetes-induced photoreceptor degeneration.

### 3.4. The Effect of AMPK on Mitochondrial Function and Autophagy in Diabetes-Induced Photoreceptor Cell Degeneration

**Figure 5:**
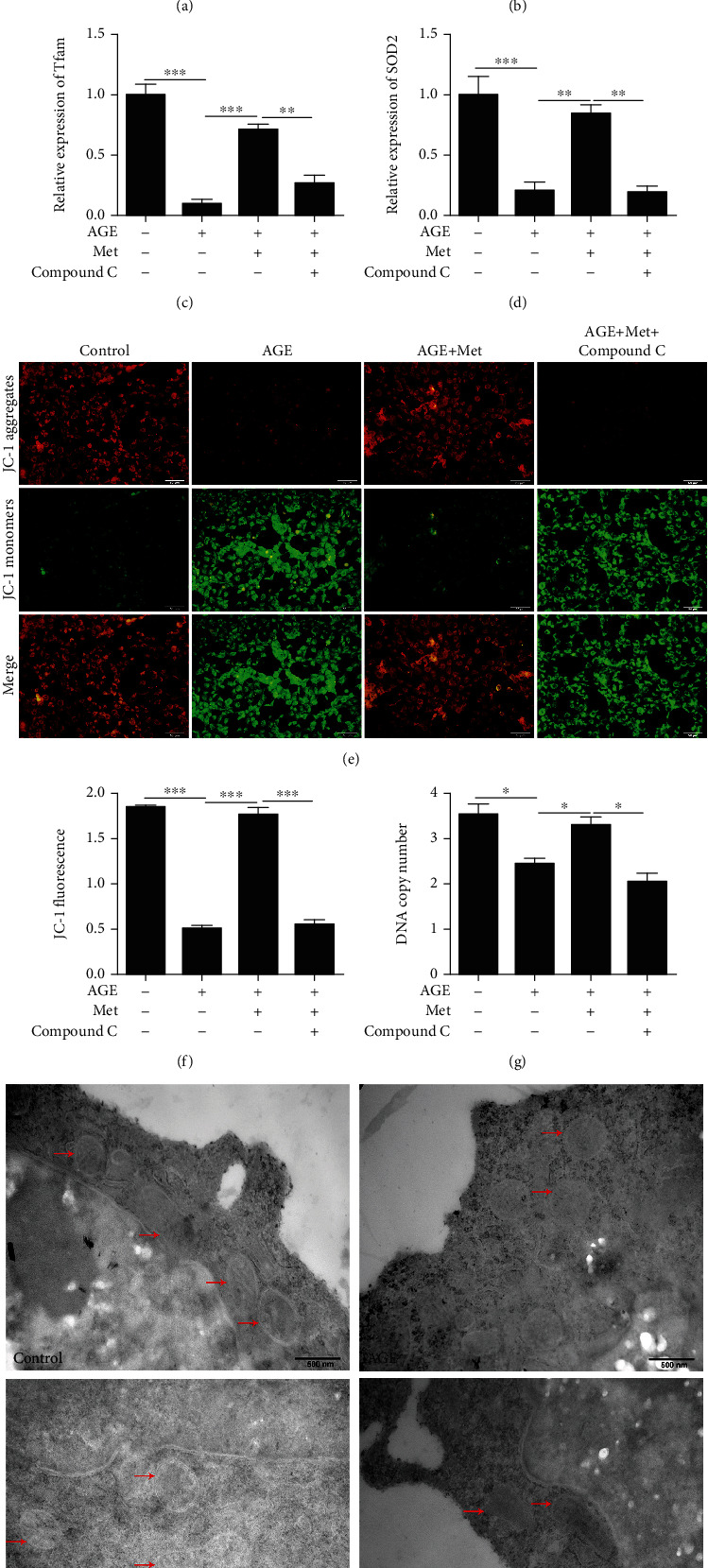
Effect of AMPK on mitochondrial function in 661w cells after AGE treatment. The effect of AMPK on the expression of PGC1*α* (a), Nrf1 (b), Tfam (c), and SOD2 (d) and the DNA copy number (g) in 661w cells treated with AGEs was measured by qRT-PCR. (e, f) The effect of AMPK on the mitochondrial membrane potential in 661w cells after AGE treatment. (h) Effect of AMPK on mitochondrial morphology in 661w cells was observed by transmission electron microscopy. The data are expressed as the mean ± SD (*n* = 3 for each group). ^∗^*p* < 0.05, ^∗∗^*p* < 0.01, ^∗∗∗^*p* < 0.001.

To confirm whether AMPK activation regulates mitochondrial function and autophagy, we treated 661w cells with the AMPK pathway inhibitor compound C. The qPCR results showed that the expression of PGC1*α*, Nrf1, Tfam, and SOD2 decreased after compound C treatment compared with that in the AMPK stimulation group (Figures 5(a)–5(d)). The mitochondrial membrane potential assay results showed that the red/green fluorescence ratio was decreased after AGE treatment in 661w cells; however, the effect was abrogated by AMPK stimulation (Figures 5(e) and 5(f)). Moreover, the results showed that the mitochondrial membrane potential and mitochondrial DNA copy number were decreased after treatment with the AMPK inhibitor compound C compared with those in the AMPK stimulation group (Figure 5(g)). Transmission electron microscopy (TEM) was used to observe mitochondrial morphology in cells. The results showed that the mitochondrial membrane and shape were unclear and abnormal compared with those in the control group. Mitochondrial morphology was improved after AMPK stimulation and inhibited by compound C treatment compared with those in the AMPK stimulation group (Figure 5(h)).

**Figure 6:**
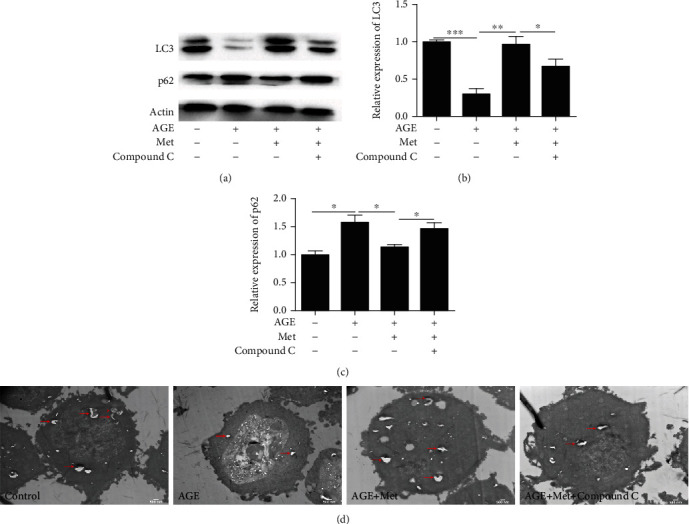
Effect of AMPK on autophagy in 661w cells after AGE treatment. Western blotting (a) was used to evaluate the effect of AMPK on the expression levels of LC3 and p62 in 661w cells treated with AGEs. Densitometric analysis showed that the elevation in LC3 (b) and p62 (c) was statistically significant. (d) Effect of AMPK on autophagosomes in 661w cells was observed by transmission electron microscopy. The data are expressed as the mean ± SD (*n* = 3 for each group). ^∗^*p* < 0.05, ^∗∗^*p* < 0.01, ^∗∗∗^*p* < 0.001.

In addition, we also measured the expression of LC3 and p62 in 661w cells after compound C treatment. The results showed that the expression of LC3 in 661w cells was increased significantly, and the expression of p62 was decreased significantly after compound C treatment compared with those in the AMPK stimulation group (Figures 6(a)–6(c)). TEM was used to observe the formation of autophagosomes. The results showed that more single- and double-membraned vesicles accumulated in the control group than in the AGE treatment group. AGEs reduced autophagy in 661w cells, while the number of autophagosomes was increased after AMPK stimulation. This effect was inhibited after compound C treatment compared with that in the AMPK stimulation group (Figure 6(d)). These results indicated that the AMPK pathway could play a vital role in regulating mitochondrial function (Figure 5) and autophagy (Figure 6) in diabetes-induced photoreceptor cell degeneration.

### 3.5. The Effect of AMPK on Diabetes-Induced Photoreceptor Cell Degeneration In Vitro

**Figure 7:**
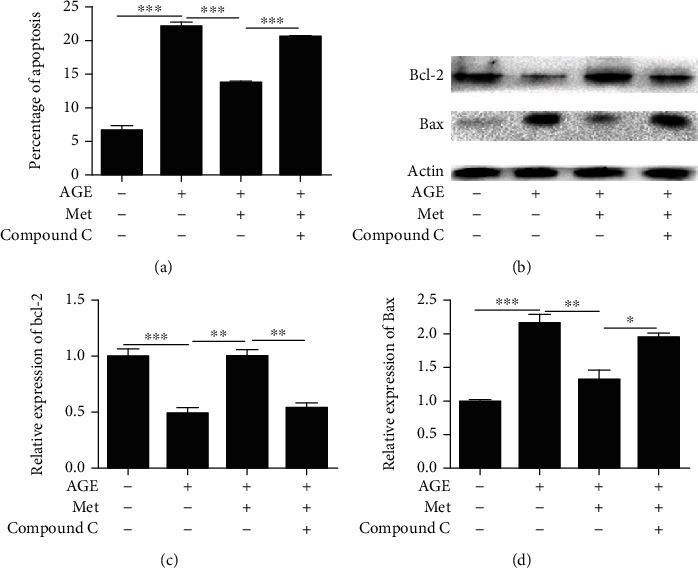
The effect of AMPK on diabetes-induced photoreceptor cell degeneration *in vitro*. (a) The percentage of apoptotic cells was analysed by DAPI staining. The expression of Bcl-2 (b, c) and Bax (b, d) in 661w cells was measured by western blotting. The data are expressed as the mean ± SD (*n* = 3 for each group). ^∗^*p* < 0.05, ^∗∗^*p* < 0.01, ^∗∗∗^*p* < 0.001.

**Figure 8:**
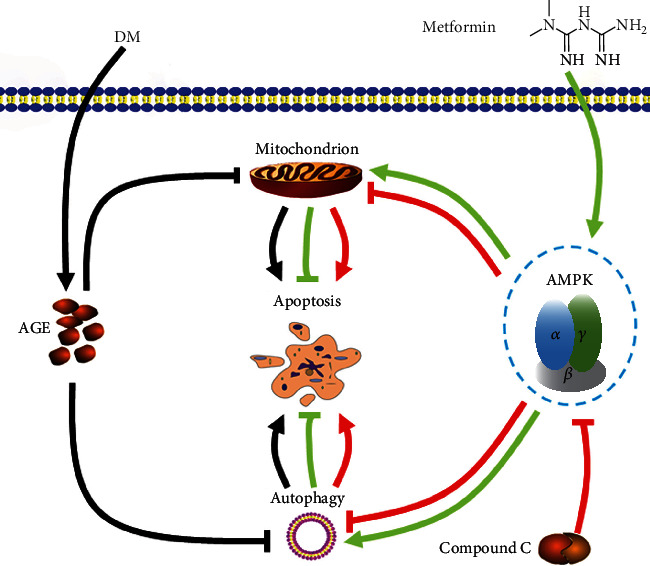
Summary of the effects and the related mechanism of AMPK stimulation on diabetes-induced photoreceptor cell degeneration.

As shown in Figure 7, the percentage of apoptotic cells was significantly increased in the AGE-treated group; however, the percentage of apoptotic cells was decreased after AMPK stimulation. This effect was suppressed by compound C (Figure 7(a)). In addition, we also showed that the expression of the apoptosis-related protein bcl-2 increased and Bax decreased after AMPK stimulation, and this effect was reversed after compound C treatment (Figures 7(b)–7(d)). These results indicate that AMPK plays a key role in AGE-induced apoptosis in 661w cells, which is summarized in Figure 8.

## 4. Discussion

661w cells are retinal-derived photosensitive cells and are often used as cell model for studying retinopathy [18]. In the present study, we used 661w cells treated with AGEs as a model to explore the regulation of AMPK on mitochondria and autophagy in the context of diabetes. AGEs can destroy the biological function of mitochondria [19] and inhibit normal autophagy in cells [20], which provides an important platform for researchers to study the molecular mechanisms and drug interventions associated with mitochondrial and autophagy-related central nervous system diseases. Some studies reported that AMPK showed protective effects on different cell lines, such as podocytes [21], hippocampal neurons [22], retinal pigment epithelium ARPE-19 cells [23], and Müller cells [24]. According to some reports, AMPK directly promotes autophagy by phosphorylating autophagy-related proteins in the mTORC1, ULK1, and PIK3C3/VPS34 complex or indirectly promotes autophagy by regulating the expression of autophagy-related genes that are downstream of transcription factors (such as FOXO3, TFEB, and BRD4). In this study, we found that AMPK stimulation significantly reversed AGE-induced apoptosis, which was consistent with previous reports. Although AMPK shows protective effects by regulating various signalling pathways, it is still unclear whether AMPK plays a neuroprotective role through mitochondria and autophagy and related mechanisms in diabetes-induced photoreceptor cell degeneration.

Mitochondria play an important role in energy metabolism. In diabetic retinopathy (DR), mitochondrial damage caused by oxidative stress is the main inducer of apoptosis [25]. AMPK can regulate mitochondrial function [26] and autophagy [27] in cells and plays an important role in the regulation of diabetes. In a high glucose environment, mitochondrial function is impaired, which leads to increased oxidative stress and changes in redox balance, thus affecting the structure and function of the retina [11].

Autophagy is a kind of catabolism. Briefly, a double-layered membrane, which detaches from the ribosome-free attachment region of the rough endoplasmic reticulum, wraps cytoplasm, organelles, and proteins that need to be degraded to form autophagosomes, which fuse with lysosomes to form autophagic lysosomes and degrade the engulfed contents to meet the metabolic needs of the cells, recycle certain organelles, and restore homeostasis [28].

In the body, normal autophagy plays an important role in regulating the steady-state function of cells [29], while abnormal autophagy is harmful [30]. Autophagy is a double-edged sword in cell injury, and its function is controversial [31]. Many studies have shown that abnormal autophagy is the fundamental and most important pathological feature of diabetic retinopathy. In addition, the complex interplay between autophagy and apoptosis plays an important role in determining the degree of apoptosis and promoting DR [32]. Increasing evidence shows that when AMPK is activated, mitochondrial function [33] and autophagy [34] are enhanced, and apoptosis is inhibited. Therefore, the regulation of mitochondria and autophagy may be helpful in preventing or treating diabetes-induced retinal photoreceptor degeneration. In our *in vitro* model, the protective effect of AMPK was verified by the enhanced expression of genes associated with mitochondrial biological function and autophagy and the increase in cell viability, which was consistent with previous data and indicated that the regulation of mitochondria and autophagy are the mechanisms of AGE-induced apoptosis.

LC3 and p62 are key autophagy proteins. LC3 is modified to form LC3I, and in the cytoplasm, LC3I is further transformed into LC3II, which is located on the autophagy membrane. p62 is an indicator of autophagy degradation products, participates in autophagy as a regulatory factor, and is degraded in the middle and late stages of autophagy. These two factors have been widely used to evaluate autophagy, and our experimental results showed that the expression of LC3II increased and the expression of p62 decreased after AMPK activation. According to the experimental results, autophagic activity increased after AMPK activation.

Some publications have revealed AMPK-mediated regulation of mitochondria and autophagy. There are several reasons for the conflicting roles of AMPK in autophagy in different studies, such as the use of different cell types and drug treatment times. In our study, in the AGE group, mitochondrial DNA copy number and the expression of related genes (PGC1a, Nrfl, TFAM, and SOD2) in 661w cells were significantly decreased compared with those in the control group, and AMPK activation significantly promoted the occurrence of mitochondrial activity, while compound C pretreatment significantly inhibited mitochondrial activity compared with that in the AMPK stimulation group. Moreover, in the AGE group, the expression of LC3 decreased significantly, and the expression of p62 increased significantly. However, the increase in p62 was effectively inhibited, and the expression of LC3 was promoted by AMPK stimulation in AGE-treated 661w cells. After pretreatment with the AMPK inhibitor compound C, this effect was reversed. Currently, many diseases have been proven to be associated with autophagy, and our results showed that AMPK stimulation protected photoreceptor cells from diabetes-induced degeneration by regulating mitochondrial biological functions and autophagy. In summary, our findings confirmed that AMPK was a key regulator of mitochondrial biological functions and autophagy in diabetes-induced photoreceptor cell degeneration and provided some evidence for the prevention or treatment of DR in the future.

## 5. Conclusion

Diabetes can increase AGE accumulation, which induces photoreceptor cell degeneration. AMPK stimulation can delay photoreceptor degeneration in diabetes by regulating autophagy and mitochondrial function to provide an effective basis for new clinical therapeutic strategies for DR.

## Data Availability

The data used to support the findings of this study are available from the corresponding author upon request.
